# A *Salmonella typhimurium* ghost vaccine induces cytokine expression *in vitro* and immune responses *in vivo* and protects rats against homologous and heterologous challenges

**DOI:** 10.1371/journal.pone.0185488

**Published:** 2017-09-29

**Authors:** Nagarajan Vinod, Han Byul Noh, Sung Oh, Seongmi Ji, Hyun Jung Park, Ki-Sung Lee, Sei Chang Kim, Han-Oh Park, Joo-Sung Yang, Chang Won Choi

**Affiliations:** 1 Department of Biology & Medicinal Science, Pai Chai University, Daejeon, Korea; 2 New Drug R&D Center, Bioneer Corporation, Daejeon, Korea; Cornell University, UNITED STATES

## Abstract

*Salmonella enteritidis* and *Salmonella typhimurium* are important food-borne bacterial pathogens, which are responsible for diarrhea and gastroenteritis in humans and animals. In this study, *S*. *typhimurium* bacterial ghost (STG) was generated based on minimum inhibitory concentration (MIC) of sodium hydroxide (NaOH). Experimental studies performed using *in vitro* and *in vivo* experimental model systems to characterize effects of STG as a vaccine candidate. When compared with murine macrophages (RAW 264.7) exposed to PBS buffer (98.1%), the macrophages exposed to formalin-killed inactivated cells (FKC), live wild-type bacterial cells and NaOH-induced STG at 1 × 10^8^ CFU/mL showed 85.6%, 66.5% and 84.6% cell viability, respectively. It suggests that STG significantly reduces the cytotoxic effect of wild-type bacterial cells. Furthermore, STG is an excellent inducer for mRNAs of pro-inflammatory cytokine (TNF-α, IL-1β) and factor (iNOS), anti-inflammatory cytokine (IL-10) and dual activities (IL-6) in the stimulated macrophage cells. *In vivo*, STG vaccine induced humoral and cellular immune responses and protection against homologous and heterologous challenges in rats. Furthermore, the immunogenicity and protective efficacy of STG vaccine were compared with those of FKC and non-vaccinated PBS control groups. The vaccinated rats from STG group exhibited higher levels of serum IgG antibody responses, serum bactericidal antibodies, and CD4^+^ and CD8^+^ T-cell populations than those of the FKC and PBS control groups. Most importantly, after challenge with homologous and heterologous strains, the bacterial loads in the STG group were markedly lower than the FKC and PBS control groups. In conclusion, these findings suggest that the STG vaccine induces protective immunity against homologous and heterologous challenges.

## Introduction

*Salmonella* are Gram-negative foodborne zoonotic bacteria that cause salmonellosis worldwide [1, 2]. *S*. *typhimurium* and *S*. *enteritidis* are mainly transmitted to humans by consumption of contaminated eggs and poultry meat products, and represent a global public health burden [3]. *Salmonella* species are composed of several serotypes that can cause infections in homologous vaccinated animals. Moreover, *S*. *typhimurium* strains are often resistant to multiple antibiotics, such as aminopenicillins, gentamicin, tetracycline, chloramphenicol, and sulfonamides [4–7]. Therefore, there is a need to develop Non-Typhoidal Salmonellae (NTS) vaccine that can induce both homologous and heterologous protective immunity against *Salmonella* strains. Meanwhile, several efforts with live attenuated vaccines have been made to induce cross-protection against NTS infections. Previous studies demonstrated that live attenuated *Salmonella aroA* [8–10], capable of reducing invasion and colonization of the gastrointestinal tract and protected animals from homologous challenge, but they lack the ability to induce heterologous protective immunity against a virulent challenge. Furthermore, the administration of attenuated *aroA* mutants from *S*. *enteritidis* [11] or *S*. *typhimurium* [12] has been shown to reduce fecal shedding against homologous challenge. However, vaccination with *Salmonella* adenylate cyclase (*cya*) and C-reactive protein (*crp*) mutant protected chickens from *Salmonella* serotypes challenges [13].

Bacterial ghosts (BG) are structurally intact empty bacterial cell envelopes prepared from various Gram-negative bacteria [14]. In BG preparation, bacterial cell lysis is induced by the controlled expression of the bacteriophage PhiX174 lysis gene *E* [15, 16] or minimum inhibitory concentration (MIC) of sodium hydroxide (NaOH) [17–21]. Based on this lysis, protein E or MIC of NaOH produces transmembrane tunnel structures on the cell surface that result in empty non-living cell envelopes. However, there are some differences in two methods. BG produced by the former method maintains their original structure, preserves all the cell surface antigens and provides efficient protection against specific infections [15–17]. Especially, functional and antigenic determinants of the envelope components are not denatured during lysis. However, there are some disadvantages of the method such as limitation to Gram-negative bacteria only, potential risks because of difficulty to reach 100% lysis rate of BG strain in a short time [22], and cost expensive and time consuming, multi-step process. Alternatively, BG produced by the latter method has no limitation to both Gram-positive [18] and Gram-negative bacteria [17,19]. This method needs a short time to generate BG without any potential risks, cheap cost and time-saving, simple process. Despite some surface structures in BG may be modified or lost by NaOH [19], the NaOH-induced BG provides efficient protection against specific infections [17,18].

In the last two decades, BG is representing to be the attractive vaccine candidate, because it can elicit both humoral and cellular immune responses against specific infections in experimental animals [23,24]. In previous studies, an effective *S*. *entritidis* and *S*. *gallinarum* ghost vaccines were produced to protect animals from *Salmonella* infections [25,26]. In the present study, non-living *S*. *typhimurium* bacterial ghosts (STG) was successfully produced by using the chemically-induced method. The main purpose of this investigation was to assess *in vitro* cytotoxicity of STG and the influence of STG on mRNA induction of cytokine and nitric oxide synthase in murine macrophages and *in vivo* protection of STG against the homologous and heterologous challenge in a rat model. Moreover, immunogenicity and protective efficacy were compared between the STG vaccine and formalin-killed inactivated cells (FKC) vaccine. This is the first study on the cross-protective efficacy of the STG vaccine in rats.

## Experimental procedures

### Bacterial strain and culture condition

*S*. *typhimurium* KCCM40253 was kindly provided by Prof. Ki-Sung Lee, Department of Biology and Medicinal Science, Pai Chai University, Daejeon, Korea. *S*. *enteritidis* was provided from an animal health product manufacturing company (KBNP, Inc., Korea). The bacteria were grown at 37°C in Luria-Bertani (LB) broth or on LB agar. Incubation temperature for growth and lysis was 37°C in a shaking incubator at 200 rpm.

### Production of *S*. *typhimurium* ghosts

STG were produced by using the MIC of NaOH as described previously [17–19]. Briefly, the MIC of NaOH against *S*. *typhimurium* was determined by the two-fold broth dilution method and 72 h cultured *S*. *typhimurium* cells were harvested by centrifugation for 10 min at 10,000 × g. After washing three times with PBS (pH 7.2), the bacterial suspension was adjusted to 1 × 10^8^ CFU/mL. One mL of the MIC of NaOH was added to 2 mL of the bacterial suspension and incubated at 37°C for 1 h. After incubation, STG were collected by centrifugation and washed three times with PBS. Samples were prepared and analyzed by scanning electron microscopy (SEM) as described previously [17,18]. For FKC vaccine preparation, 0.5% formalin was added to the bacterial suspension and incubated for 24 h at 37°C. After incubation, the bacterial suspension was washed three times with PBS, and the bacterial suspension was plated on LB agar plates to confirm the total loss of viability. FKC was stored at 4°C until use.

### Assessment of macrophage-mediated cytotoxicity

Murine macrophage (KCLB:40071, RAW 264.7) cells were purchased from Korean Cell Line Bank (Seoul, Korea) and cultured in 96-well plates (BD Falcon; BD Bioscience Discovery Labware, Bedford, MA, USA) for 24 h at 37°C, in humidified 5% CO_2_, 95% air. The cells (1.0 × 10^4^ cells/well) were then treated with 1.0 × 10^8^ CFU/mL of the FKC, wild-type bacterial cells and NaOH-induced STG, respectively, in culture medium, and incubated for a further 24 h. PBS treated- and LPS-treated macrophages were used as negative and positive controls, respectively. LPS was extracted from *S*. *typhimurium* (1 × 10^8^ CFU/mL) using an LPS extraction kit (iNtRON Biotechnology Inc., Seongnam-si, Gyeonggi-do, Korea) according to the manufacturer’s protocol. Briefly, 5 mL culture was centrifuged at 13,000 rpm for 5 min. The bacterial pellet was treated with 1 mL of supplied lysis buffer and vortexed vigorously. After adding 200 μL of chloroform, the mixture was centrifuged at 13,000 rpm for 10 min at 4°C. Then, the supernatant (400 μL) was mixed well with a supplied purification buffer and incubated for 10 min at −20°C. After centrifuging the mixture solution at 13,000 rpm for 15 min at 4°C, the upper layer was removed to obtain the LPS pellet. The pellet was washed with 70% ethanol and centrifuged at 13,000 rpm for 3 min at 4°C. After discarding the resulting upper layer, the pellet was dried at room temperature and dissolved in 10 mM Tris-HCl (pH 8.0) by boiling for 2 min. To get the pure LPS, proteinase K (2.5 μg/ LPS 1 μg) as added to the dissolved LPS and the mixture was incubated at 4°C for 30 min. The cell density was then assessed by using Cell Counting Kit-8 (CCK-8, Sigma-Aldrich, St. Louis, MO, USA). Absorbance was measured at 450 nm and all experiments were performed in triplicate. Cytotoxic activity is expressed as the percentage of cell viability by the following formula: % Cytotoxicity = (1 − A_450nm_ of target cells/A_450nm_ of control cells) × 100.

### Quantitative analysis of cytokine mRNA by reverse transcription (RT)-qPCR

RAW 264.7 cells (1.0 × 10^4^ cells/well) were cultured in 24-well flat-bottom plates and treated with the FKC, wild-type bacterial cells and NaOH-induced STG, respectively, at a concentration of 1.0 × 10^8^ CFU/mL. After 1–12 h stimulation, total RNA was isolated using RNAiso (Takara Bio, Shiga, Japan), according to the manufacturer’s instructions. Tumor necrosis factor (TNF)-α, interleukin (IL)-1β, IL-6, IL-10 and inducible nitric oxide synthase (iNOS) mRNA levels were quantified by RT-qPCR amplification. Sequences for the primers of target genes are listed in S1 Table. RT reaction was performed in a 20 μL reaction mixture containing 300 ng of total RNA, 50 mM Tris-HCl (pH 8.3), 75 mM KCl, 8 mM MgCl_2_, 10 mM DTT, 0.1% NP-40, 40 mM dNTP, 2 pM of respective primer set, 20 U of RNase inhibitor (Takara Bio), and 200 U PrimeScript Reverse Transcriptase (Takara Bio). The thermal cycler was programmed for 1 RT cycle at 50°C for 30 min and 70°C for 15 min. cDNA was amplified in a 20 μL reaction mixture containing 10 μL 2X SYBR^®^ Premix Ex Taq™ II (Tli RNaseH Plus, Takara Bio, Shiga, Japan), 0.2 μL ROX reference dye II, 0.4 μL of 10 μM of both forward and reverse primer and 1 ng of cDNA, using Stratagene Mx3005P cycler (1 cycle at 95°C for 30 s, 30 cycles of denaturation at 95°C for 5 s, and primer annealing and extension at 60°C for 34 s). Each gene was amplified in triplicate and cDNA concentration differences were normalized to glyceraldehyde 3‐phosphate dehydrogenase (GAPDH).

### Experimental animals

Ten-week-old male Sprague-Dawley rats were housed under standard conditions of temperature and relative humidity with a 12 h light/dark cycle. Food and water were available *ad libitum*. All animal experiments were approved by institutional animal care and use committee at Pai Chai University.

### Vaccination and challenge protocol

Thirty-nine rats were equally divided into three groups: A, B, and C. The rats in group A (n = 13) were injected subcutaneously with sterile PBS as a non-vaccinated control. The rats in groups B (n = 13) and C (n = 13) were vaccinated subcutaneously with FKC (1 × 10^8^ cells/mL) and STG (1 × 10^8^ cells/mL), respectively. Rats from all groups were vaccinated three times at two-week intervals. Two weeks after the final vaccination (week 7), all rats were challenged orally with *S*. *typhimurium* (1 × 10^8^ CFU/mL) [27] or *S*. *enteritidis* (2 × 10^6^ CFU/mL) in PBS [17,28,29].

### Detection of antibody levels by ELISA

Serum Immunoglobulin (Ig) G antibodies were determined by indirect enzyme-linked immunosorbent assay (ELISA) as previously described [18,19]. Microtiter plates were coated with 100 μL of *S*. *typhpimurium* or *S*. *enteritidis* (1 × 10^8^ cells/mL) in coating buffer (pH 9.6). ELISA was carried out with sera from non-vaccinated and vaccinated rats. Goat anti-rat IgG conjugated alkaline phosphatase (1:30,000; Sigma-Aldrich) was used as the secondary antibody. The plates were developed with 100 μL of the substrate p-nitrophenyl phosphate (Sigma-Aldrich, St. Louis, MO) and 100 μL of 3N NaOH was added to stop the reaction. The optical density was measured at 405 nm with a microplate reader (Bio-Rad, USA).

### Analysis of T-cells populations by FACS

To analyze T cell populations, blood samples were collected from non-vaccinated and vaccinated rats at one week after the final vaccination. The peripheral blood mononuclear cells were prepared using Histopaque-1077 (Sigma-Aldrich, St. Louis, MO) according to the manufacture’s protocol. The cells (1.0 × 10^6^) were washed three times in PBS and incubated either fluorescein isothiocyanate (FITC) labeled anti-CD4 or CD8 monoclonal antibodies at 4°C for 30 min in the dark. After the incubation, all the samples were washed twice with PBS, and analyzed on a BD FACSCanto II flow cytometer (BD Biosciences, NJ, USA). Data analysis was done on BD FACSDiva software (BD Biosciences).

### Determination of serum bactericidal activity

The bactericidal activity of the sera was tested as described previously [30]. Twenty-five μL of serum were mixed with 100 μL of *S*. *typhimurium* (1 × 10^6^ CFU/mL) or *S*. *enteritidis* (1 × 10^6^ CFU/mL) and incubated for 1 h at room temperature. After incubation, samples were plated on selective media and incubated for 24–48 h at 37°C. Instead of serum, PBS was served as a control. The percentage of serum bactericidal activity was determined by using the following formula: SBA = {1 –(the number of viable bacteria after serum treatment/the number of viable bacteria after PBS treatment)} × 100%.

### Western blot analysis

*S*. *typhimurium* or *S*. *entertidis* envelope proteins were extracted as previously described [31,32]. Twenty μg of protein were loaded and subjected to 12% Sodium dodecyl sulfate-polyacrylamide gel electrophoresis (SDS-PAGE). Western blots were probed with vaccinated or non-vaccinated sera (1:1,000) in non-fat dry milk as primary antibodies. Goat anti-rat IgG conjugated alkaline phosphatase (1:7,000; Sigma-Aldrich) was used as a secondary antibody. Detection was developed using nitroblue tetrazolium and 5-bromo-4-chloro-3-indolylphosphate (MBI Fermentas, Canada).

### Bacterial clearance analysis

To evaluate the STG vaccine-induced protective immunity against homologous and heterologous challenges, the rats were sacrificed on 2 weeks post-challenge. The liver, lung, spleen, and kidney of each was harvested aseptically and homogenized in PBS using a tissue homogenizer (Sun MI Technology, Korea). Serial dilution of homogenates was then plated on selective media and incubated at 37°C for 24–48 h. After incubation, CFUs were determined.

### Statistical analysis

All statistical analyses were performed with one-way ANOVA using SPSS software (version 14.0) and data were expressed as mean ± standard error of the mean. Differences were considered statistically significant at *P* values of < 0.001, < 0.01 and < 0.05.

## Results

### Production and characterization of *S*. *typhimurium* ghosts

**Fig 1:**
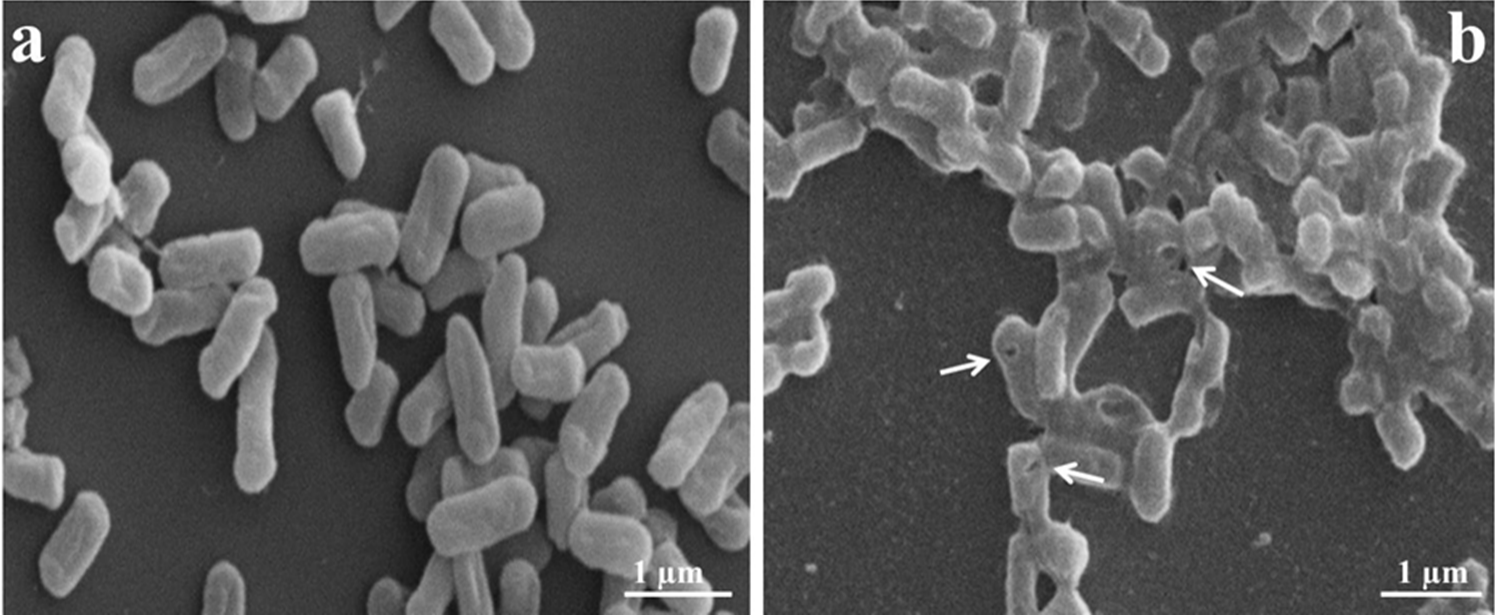
**Scanning electron microscopic analysis of (a) *S*. *typhimurium* and (b) STG.** Arrows indicate trans-membrane lysis tunnels.

In order to produce STG, we first determined the MIC of NaOH (3.13 mg/mL) against *S*. *typhimurium* by using the two-fold broth dilution method. STG was successfully produced by using the specific concentration of NaOH. At the end of lysis process, no viable colonies were detected in the MIC of NaOH-induced STG. This indicates that the efficiency of chemically- induced STG was 100%. Formation of the transmembrane tunnels in STG was examined by SEM (Fig 1A and 1B). Electron microscopic analysis revealed the presence of transmembrane lysis tunnel structures in STG (Fig 1B) as compared to the unlysed wild-type *S*. *typhimurium* cells (Fig 1A). Although the cellular morphology doesn’t seem to be affected by the MIC of NaOH, some of the cell surface structures seem to be slightly affected.

### Cytotoxicity tests and induction of cytokine mRNA expression in murine macrophages exposed to STG

**Fig 2:**
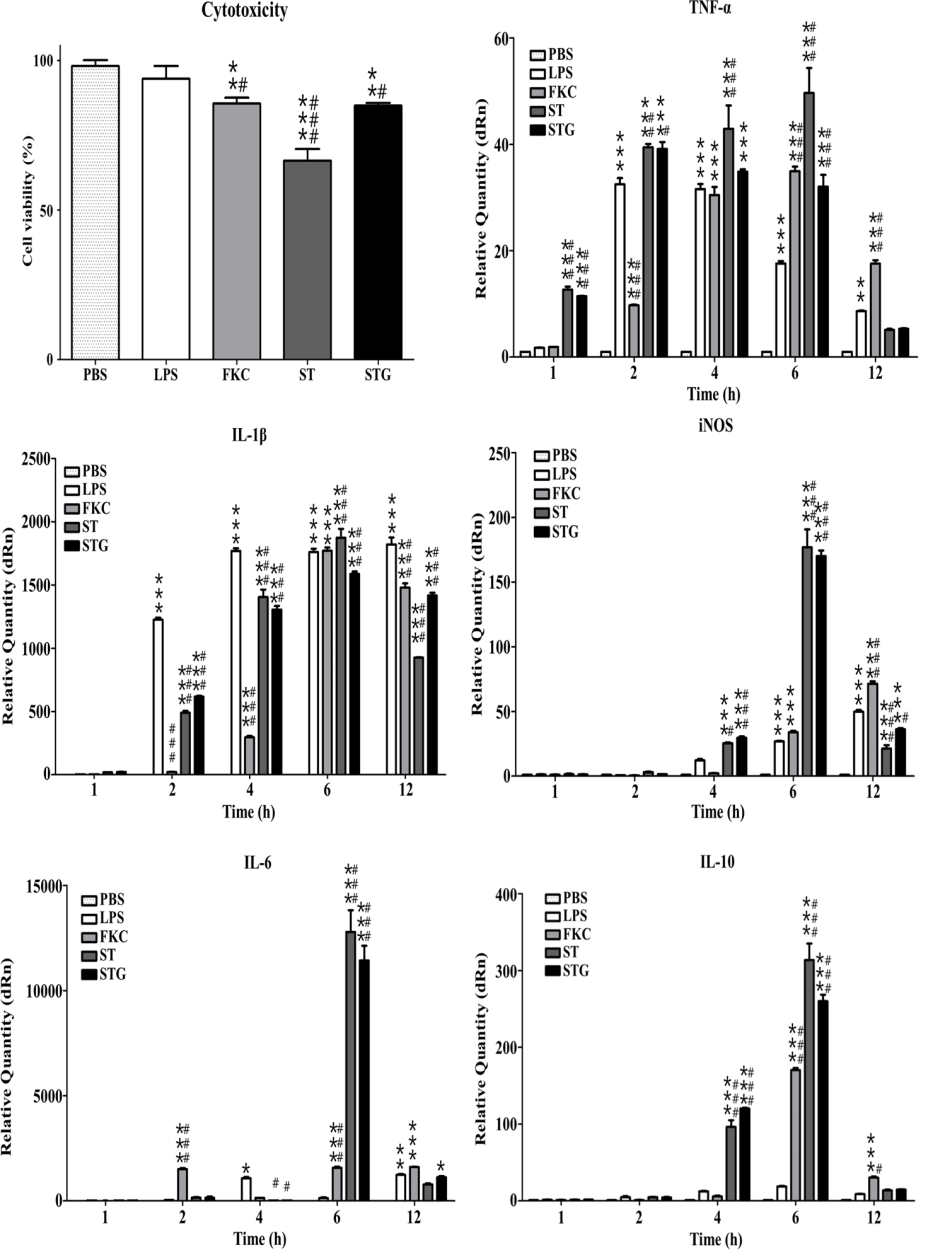
Cell viability and pro‐ and anti‐inflammatory cytokine production of STG. To determine cytotoxicity, murine macrophages (RAW 264.7) were exposed to PBS buffer, LPS purified from *S*. *typhimurium*, FKC, wild-type bacterial cells (ST) and NaOH-induced STG, respectively (bars). At 24 h post-exposure, macrophages were collected for analysis of cell viability using Cell counting Kit-8. Absorbance was measured at 450 nm and all experiments were performed in triplicate. Cytotoxic activity is expressed as the percentage of cell viability by the formula described in Materials and Methods. At given time post‐exposure with PBS buffer, LPS purified from *S*. *typhimurium*, FKC, ST and STG, respectively (bars), macrophages were collected for analysis of mRNA expression for pro- and anti-inflammatory cytokines and factor (TNF‐α, IL‐1β, iNOS, IL‐6 and IL‐10) using RT-qPCR. Data are representative of triplicate experiments with each sample run in triplicate. All statistical analyses were performed with one-way ANOVA using SPSS software (version 14.0) and data were expressed as mean ± standard error of the mean. *** = *P* < 0.001; ** = *P* < 0.01; * = *P* < 0.05 (significant difference from PBS) and ### = *P* < 0.001; ## = *P* < 0.01; # = *P* < 0.05 (significant difference from LPS).

The cytotoxicity was compared using the viability of RAW 264.7 murine macrophages exposed to the FKC, wild-type bacterial cells and NaOH-induced STG, respectively (Fig 2). The given concentration of *S*. *typhimurium* LPS included in this analysis showed cell viability of 93.9%. The macrophages exposed to STG showed 84.9% cell viability (PBS: *P* < 0.01 and LPS: *P* < 0.05), which was similar to those exposed to FKC (85.6%) but significantly higher than those exposed to wild-type bacterial cells (66.5%) under the same concentration.

To determine the activation of macrophages exposed to STG to produce inflammatory cytokines, mRNA expression of pro-inflammatory cytokines and factor (TNF-α, IL-1β and iNOS), anti-inflammatory cytokine (IL-10) and dual properties of cytokine (IL-6) was investigated using RT-qPCR (Fig 2). In TNF-α mRNA, the highest mRNA level was found at 6 h in the macrophages exposed to wild-type bacterial cells (PBS and LPS: *P* < 0.001), which was much higher than those exposed to FKC and STG, respectively. In STG-exposed macrophages, the maximum accumulation of TNF-α mRNA was found at 2 h (PBS: *P* < 0.001 and LPS: *P* < 0.05), which was a similar quantity found in macrophages exposed to wild-type bacterial cells, and then declined in a time-dependent manner. Similarly, the induction of IL-1β mRNA was induced in a time-dependent manner in the macrophages exposed to STG. Its maximum level was found at 6 h, which was less than those exposed to FKC and wild-type bacterial cells. In iNOS, IL-6 and IL-10 mRNA, the respective maximum levels were found at 6 h in the macrophages exposed to wild-type bacterial cells (PBS and LPS: *P* < 0.001), which was slightly higher than those exposed to STG. Taken together, our data indicated that STG can activate macrophages to secrete both pro-inflammatory and anti-inflammatory cytokines.

### Humoral immune response

**Fig 3:**
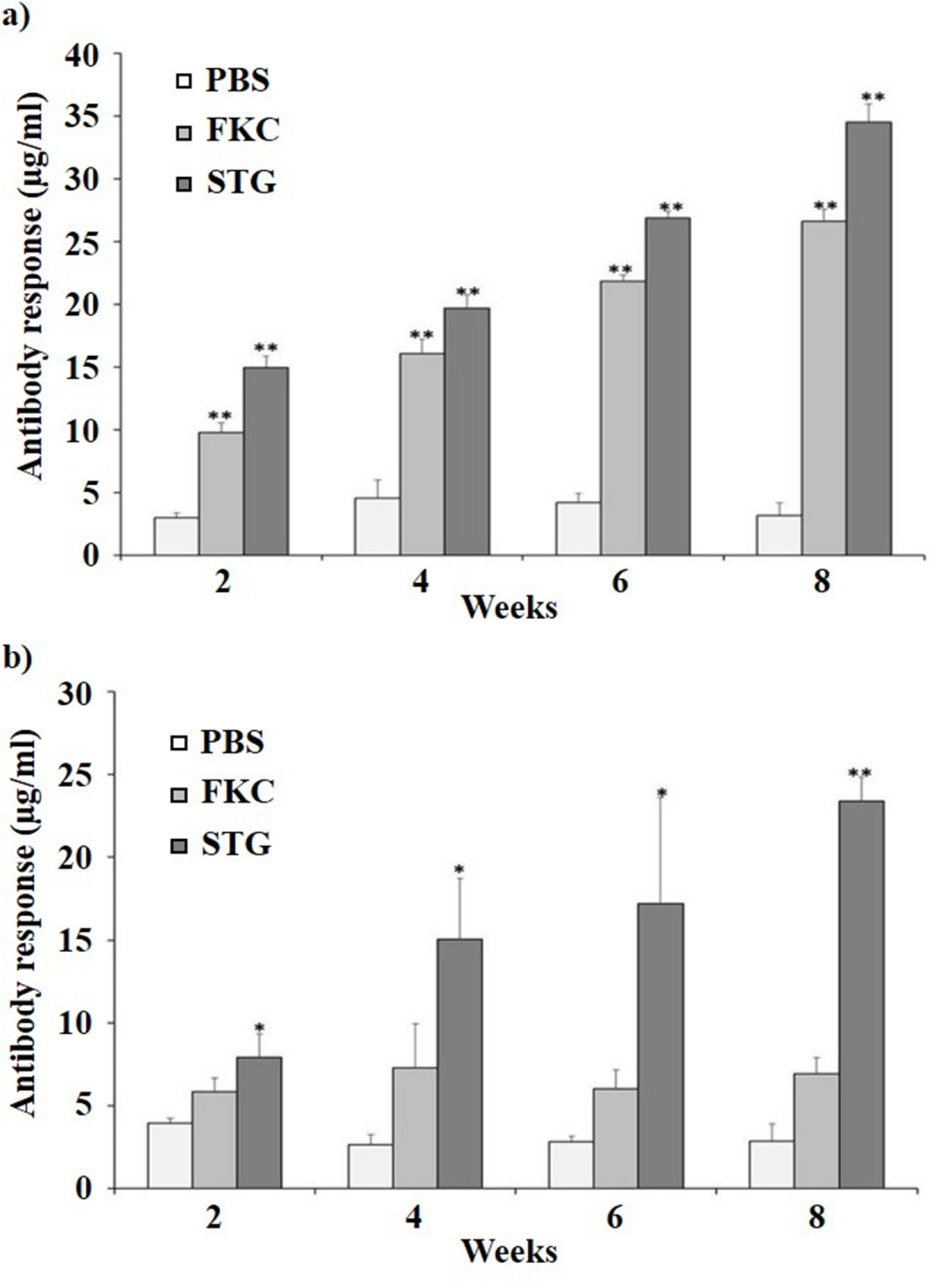
The levels of IgG antibody responses were determined by indirect ELISA. (a) *S*. *typhpimurium* and (b) *S*. *enteritidis* were coated as an antigen for ELISA. Results are expressed as means ± standard errors of the means. The asterisks indicate significant differences between antibody responses of the vaccinated and non-vaccinated groups. ** = *P* < 0.01; * = *P* < 0.05.

To evaluate humoral immune responses of the STG vaccine against *S*. *typhimurium* and *S*. *enteritidis* antigens, the titers of total IgG in the sera of vaccinated and non-vaccinated animals were determined by indirect ELISA (Fig 3). Rats from FKC and STG groups elicited higher serum IgG antibody responses as compared to the PBS control group (Fig 3A). However, there were significant differences in the levels of serum IgG antibodies between FKC and STG at weeks 2 and 6. At week 9 post-challenge, rats vaccinated with either STG or FKC showed significantly higher IgG antibody responses than PBS control rats (*P* < 0.01). Most importantly, serum IgG antibodies in STG group rats increased robustly as compared to PBS group (*P* < 0.01) and FKC group (*P* < 0.05) throughout the vaccination and *S*. *enteritidis* challenge period (Fig 3B).

### Cellular immune response

**Fig 4:**
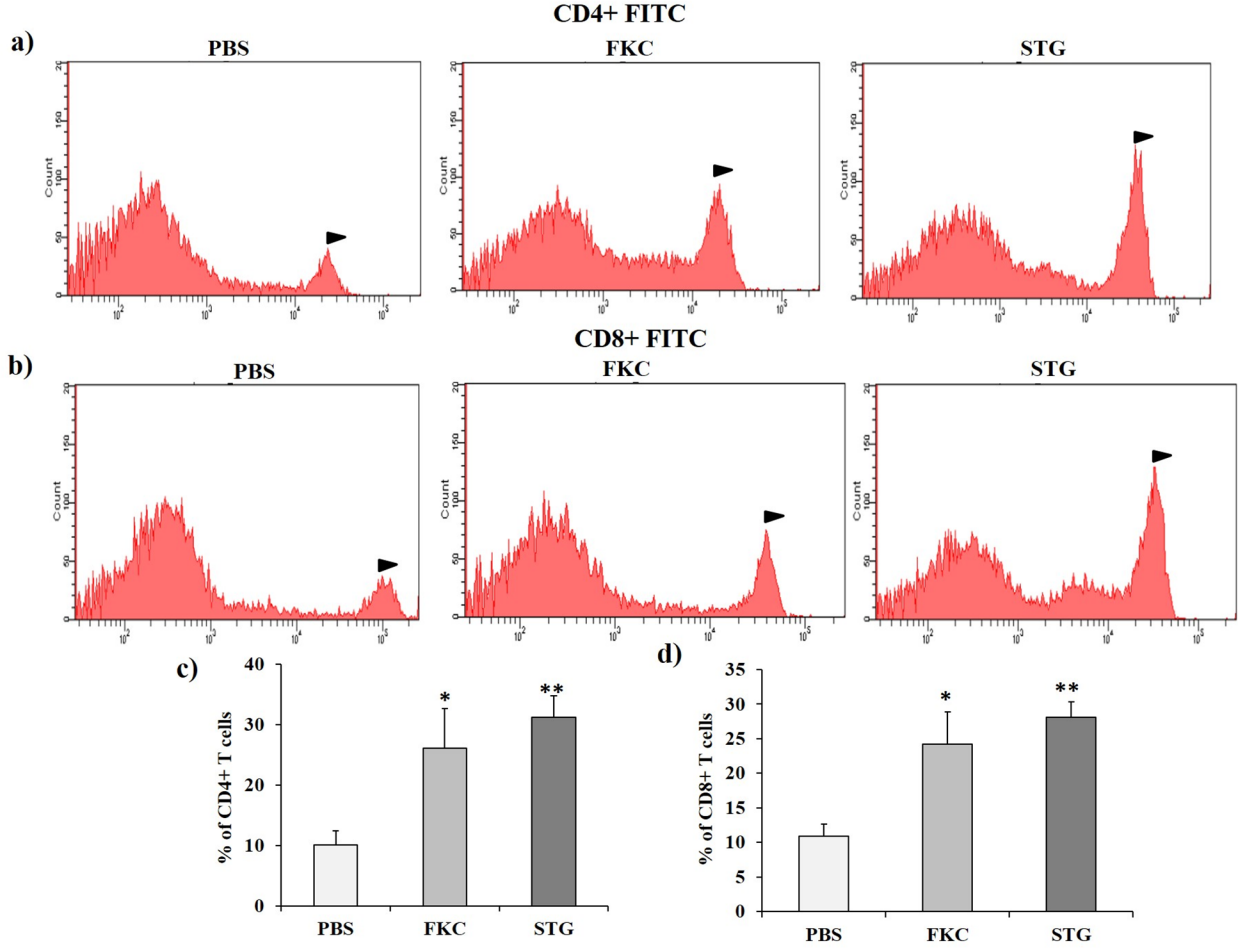
Assessment of CD4^+^ and CD8^+^ T-cells by FACS analysis. Populations of (a) CD4^+^ and (b) CD8^+^ T-cells and (c and d) the corresponding significant analysis from vaccinated and non-vaccinated control groups one week (week 6) after the final vaccination with STG. Values are shown as means ± standard errors of the means. The asterisks indicate significant differences between T-cell populations of the vaccinated and non-vaccinated groups. ** = *P* < 0.01; * = *P* < 0.05.

The CD4^+^ and CD8^+^ T-cell populations in vaccinated and non-vaccinated animals were observed by FACS (Fig 4A and 4B). As shown in Fig 4C, CD4^+^ T-cell populations significantly increased in all vaccinated animals (FKC: *P* < 0.05 and STG: *P* < 0.01) than the non-vaccinated control PBS group, but no significant difference detected between FKC and STG. Similarly, animals from FKC (*P* < 0.05) and STG (*P* < 0.01) had statistically higher percentage of CD8^+^ T-cells compared to the control PBS group (Fig 4D). Meanwhile, the statistical difference between FKC and STG was not significant.

### Determination of serum bactericidal activity

**Fig 5:**
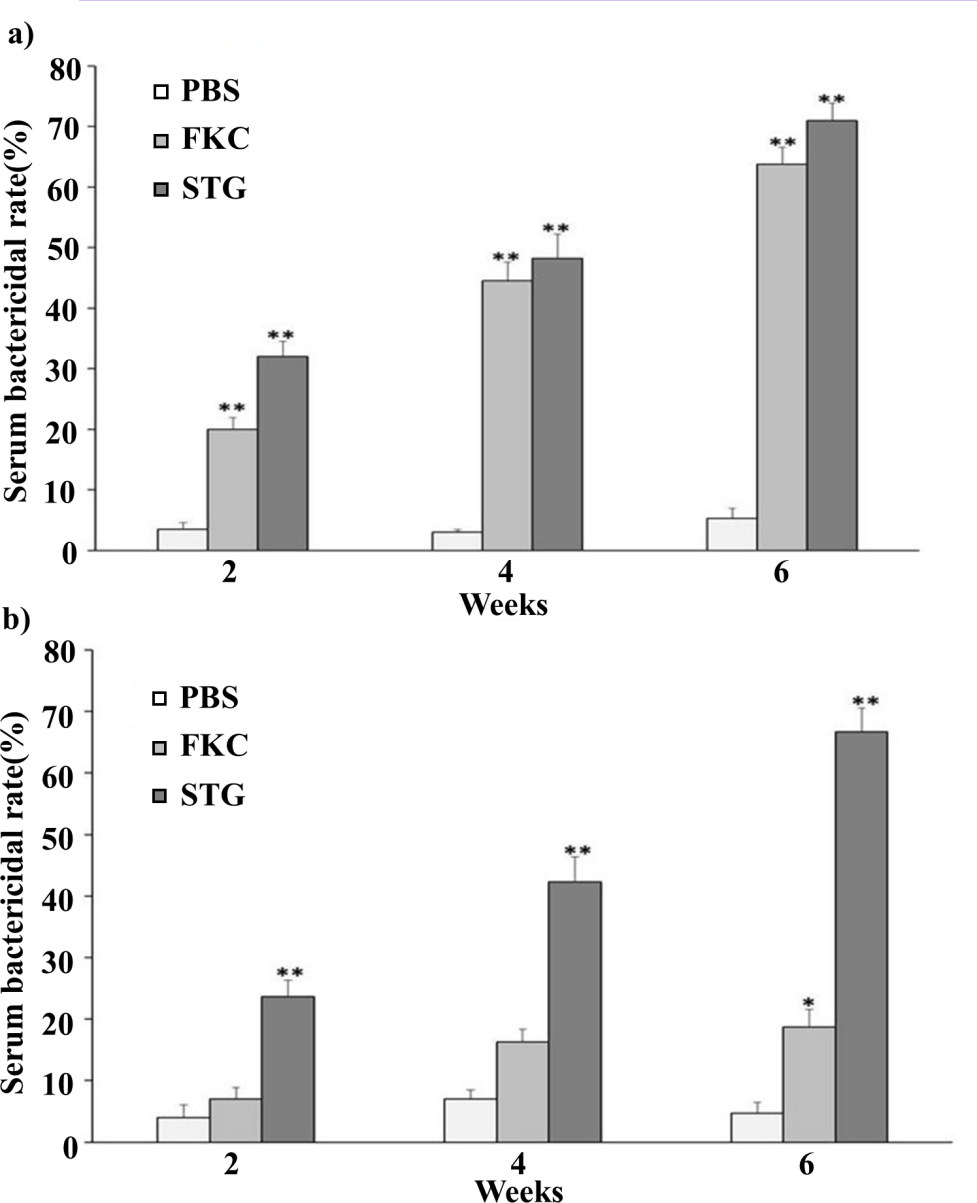
**Serum bactericidal activities of rats vaccinated with STG against (a) *S*. *typhpimurium* or (b) *S*. *enteritidis*.** Data were expressed as means ± standard errors of the means. The asterisks indicate significant differences between serum bactericidal activities of the vaccinated and non-vaccinated groups. ** = *P* < 0.01; * = *P* < 0.05.

Serum bactericidal activity from rats vaccinated with the STG vaccine was tested against *S*. *typhimurium* (Fig 5A) and *S*. *enteritidis* (Fig 5B). As can be seen in Fig 5, the group vaccinated with the STG vaccine showed significantly higher levels of bactericidal antibodies compared to FKC and PBS control groups (*P* < 0.01). However, at weeks 4 and 6, bactericidal activity was not significantly different between STG and FKC against homologous strain. Interestingly, FKC vaccinated rats showed significant bactericidal antibodies against heterologous strain at week 6 (*P* < 0.05). Overall, the results suggest that STG vaccinated group increased bactericidal activities throughout the vaccination period (weeks 2, 4, and 6).

### Immuno-blotting examination of the antibody response

**Fig 6:**
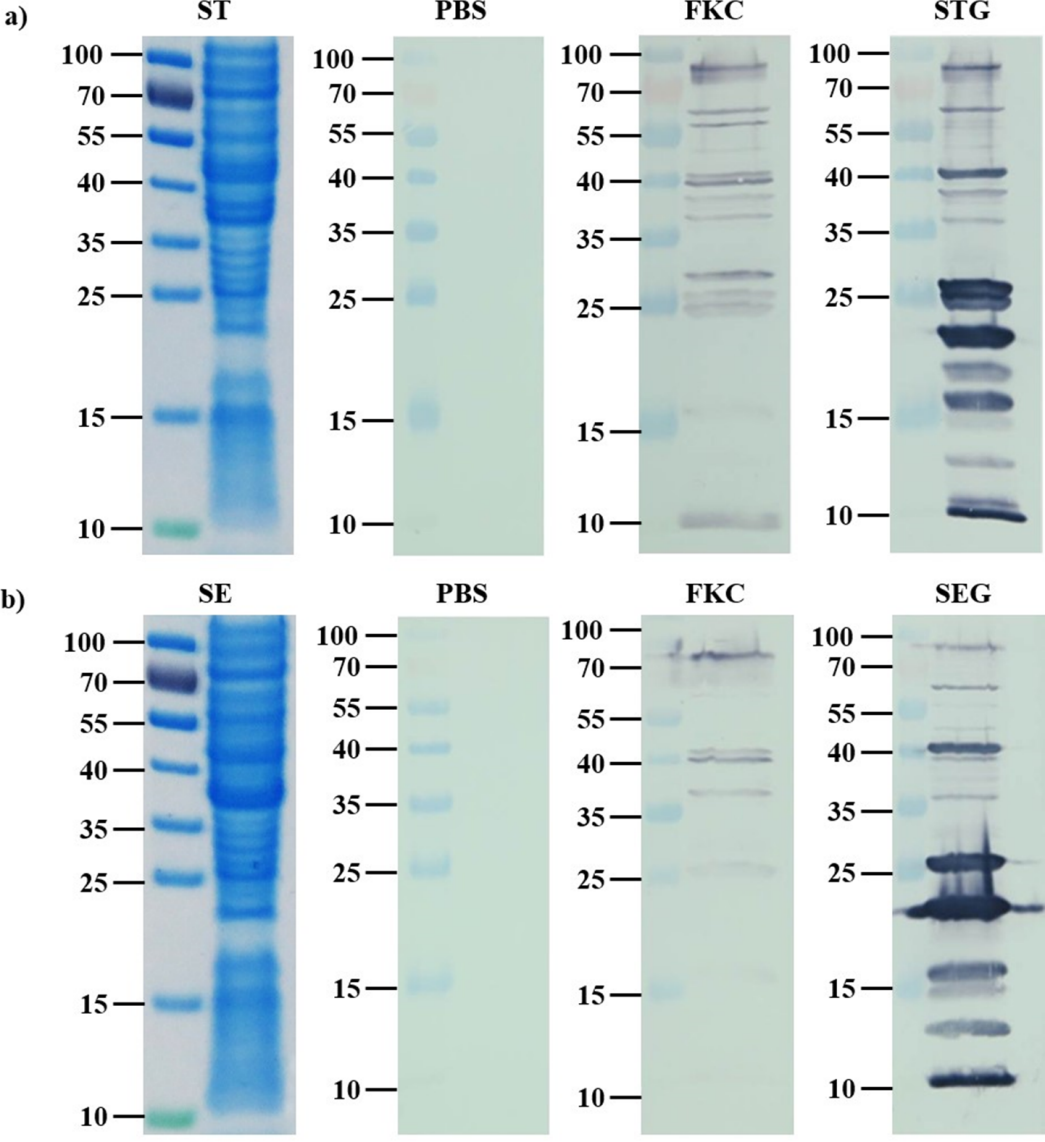
**Western blot analysis of envelope proteins from (a) *S*. *typhimurium* or (b) *S*. *enteritidis* probed with PBS, FKC and STG sera.** Coomassie Brilliant Blue-stained SDS-PAGE (12%) gels containing envelope proteins of *S*. *typhimurium* (ST) or *S*. *enteritidis* (SE) are in the left side of (a) and (b) panels.

Specific anti-*S*. *typhimurium* and anti-*S*. *enteritidis* antibody responses in sera of vaccinated and non-vaccinated control animals were further analyzed by immunoblot using *S*. *typhimurium* or *S*. *enteritidis* envelope proteins as antigens. Serum from STG vaccinated animals were capable of recognizing envelope protein antigens from *S*. *typhimurium* and showed stronger protein bands including OMPs and envelope proteins than other groups (PBS and FKC). The molecular masses of envelope protein antigens were approximately 82, 62, 59, 57, 50, 40, 38, 37, 36, 27, 25, 21, 19, 17, 15, 12, 11 and 10 kDa (Fig 6A). Similarly, envelope proteins from *S*. *enteritidis* were able to cross-recognize by STG vaccinated serum. The molecular masses of *S*. *enteritidis* envelope protein antigens were found to be approximately 82, 62, 55, 47, 40, 38, 37, 36, 27, 21, 16, 15, 13 and 10 kDa (Fig 6B). Analysis of sera from FKC vaccinated animals showed weaker bands against *S*. *enteritidis* protein antigens.

### Homologous and heterologous protections of *S*. *typhimurium* ghost vaccine

**Fig 7:**
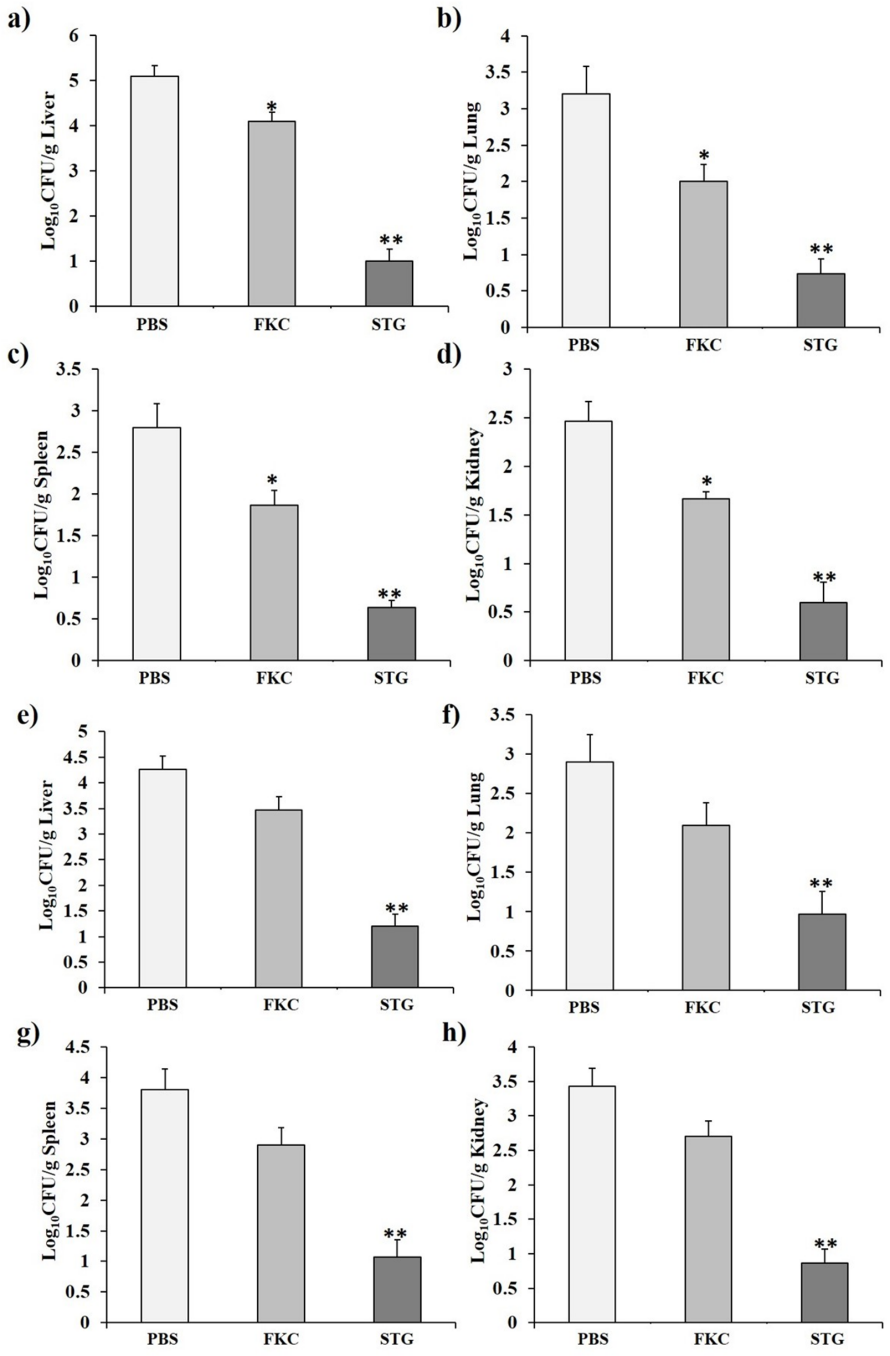
Protection of STG against homologous and heterologous challenges. Bacterial loads in liver, lung, spleen and kidney homogenates after (a-d) a homologous challenge with *S*. *typhimurium* or (e-h) a heterologous challenge with *S*. *enteritidis*. Results are expressed as means ± standard errors of the means. The asterisks indicate significant differences between the bacterial clearance of the vaccinated and non-vaccinated groups. ** = *P* < 0.01; * = *P* < 0.05.

To examine the ability of the STG vaccine against *S*. *typhimurium* challenge, rats were challenged orally with *S*. *typhimurium* two weeks after the final booster vaccination. The bacterial loads in the liver, lung, spleen, and kidney were significantly lower in FKC (*P* < 0.05) and STG (*P* < 0.01) than in PBS (Fig 7A–7D). The protection against *S*. *typhimurium* afforded by the STG vaccine was significantly greater than that afforded by the FKC vaccine (*P* < 0.05). In order to determine the cross-protective efficacy of the STG vaccine, rats were vaccinated with STG, FKC or PBS and challenged with a virulent *S*. *enteritidis* strain. The results of the bacterial loads in liver, lung, spleen, and kidney are shown in Fig 7E–7H. The bacterial count in liver, lung, spleen, and kidney were significantly lower in STG than those in PBS and FKC (*P* < 0.05). These results suggest that the vaccination with STG vaccine provided protection against *S*. *enteritidis* strain, but FKC vaccinated rats failed to protect against *S*. *enteritidis* strain.

## Discussion

The development of safe and effective vaccines against different *Salmonella* serotypes is an urgent need for the control and prevention of *Salmonella* infections. It is known that macrophages and dendritic cells are the key cells to be infected by *S*. *typhimurium*, and these antigen-presenting cells transport the bacteria to the liver and spleen, the major organs of bacterial replication [33,34]. Both cells are critical components to the immune response to *S*. *typhimurium* infection, with macrophages controlling bacterial growth in the early phase of infection and dendritic cells initiating the T cell response [35–37].

In this study, the higher cell viability in the macrophages exposed STG than that of wild-type bacterial cells, suggesting that the MIC of NaOH reduces the cytotoxic effect of wild-type bacterial cells. Previously, alkaline treatment to a Gram-negative bacterial LPS resulted in reduced toxicity and deacylated LPS. The resultant LPS showed intact amide-linked fatty acids and removal of ester-linked fatty acids [38], but it was antigenically poor [38,39] or deficient [40]. However, our previous studies demonstrated that immunization with the NaOH-induced BG from a Gram-negative bacterium or a Gram-positive bacterium induces effective immune responses and provides a good protection against virulent challenge [17,18]. The present study also supports previous studies and suggests that the NaOH treatment does not affect the immunogenicity of BG or its potential as a vaccine candidate. Furthermore, our previous results showed that LPS of BG is modified or party lost by NaOH treatment and suggested that immunogenicity of the NaOH-induced BG could be derived from other cell envelope components rather than LPS [19].

Murine macrophages respond with secreting a variety of cytokines (TNF-α, IL-1, IL-6, IL-10 and IL-12) [37,41], chemokines, cell surface receptors, signaling molecules and transcriptional activators [42]. Simultaneously, co-incubation *S*. *typhimurium* with murine macrophages enhanced production of oxygen-dependent antmcrobial molecules such as superoxide, hydrogen peroxide and nitric oxide (NO) to kill intracellular *Salmonella* bacteria [43–45]. Like wild-type *S*. *typhimurium*, the NaOH-induced STG effectively activated the mRNA expression of TNF-α, IL-1β, iNOS, IL-6 and IL-10 in this study.

Furthermore, we demonstrate that the NaOH-induced STG vaccine has the ability to induce both humoral and cellular immune responses and protects rats from *S*. *typhimurium* and *S*. *enteritidis* challenges. The cellular morphology and cell surface structures were unaffected by the MIC of NaOH during lysis process. This result indicates that the MIC of NaOH successfully induced non-living STG vaccine. Previously, we produced the chemically induced BG from a Gram-positive bacterium *Staphlococcus aureus* and a Gram-negative bacteria *S*. *enteritidis* and *Vibrio parahaemolyticus* with 100% lysis efficiency [17–20].

In our previous study with chemically-induced *S*. *enteritidis* ghosts (SEG), the SEG vaccine has shown the usefulness of detecting serum IgG antibody response after challenge and provided the protective immunity that might be mediated by induction of humoral immune responses [17]. Peng et al [46] also demonstrated that humoral immune responses play an important role in preventing *Salmonella* infections. In this regard, serum IgG antibodies in STG vaccinated rats after challenge with *S*. *typhimurium* and *S*. *enteritidis* strains were evaluated. Compared with the levels of antibody response in PBS and FKC control groups, STG vaccinated animals had higher serum IgG antibody response throughout the vaccination as well as the challenge period. Similar results were shown in *S*. *enteritidis* challenge studies, suggesting STG vaccine has the ability to induce protective immune responses against different *Salmonella* serotypes. In contrast, the FKC vaccine induced significant protective immunity against the *S*. *typhimurium* strain, but failed to induce significant antibody responses and protective immunity against the *S*. *enteritidis* strain.

Western blot analysis supported the difference between immune response induced by the STG vaccine and FKC vaccine. Compared with the FKC vaccine, the STG vaccinated sera showed stronger protein bands against the *S*. *typhimurium* and *S*. *enteritidis* antigen. This could be due to the loss of major surface antigenic components in the FKC vaccine. Hofstra et al [47] described that antigenic cross-reactivity of the major outer membrane proteins (30–42 kDa) was a general phenomenon in Enterobacteriaceae. The lack of heterologous antibodies revealed in the western blot analysis could be a reason for the lesser protection against the *S*. *enteritidis* challenge. In both the *S*. *typhimurium* and *S*. *enteritidis* strains, the serum bactericidal antibodies were significantly induced in STG vaccinated animals as compared to FKC and PBS vaccinated animals. Particularly, serum bactericidal analysis revealed that rats vaccinated with FKC vaccine produced higher levels of antibodies to the *S*. *typhimurium* strain. These antibodies could not induce cross-protective antibodies to the *S*. *enteritidis* strain. The data from the present study indicate that vaccination with the STG vaccine induces a strong protective immunity against *S*. *typhimurium* and *S*. *enteritidis* challenges.

It has been reported that BG from pathogenic bacteria has the ability to induce strong humoral and cellular immune responses and protect experimental animals from virulent challenge [48,49]. CD4^+^ MHC II- and CD8^+^ MHC I-restricted T cells play an important role in destroying intracellular bacteria, such as *Salmonella* [50–52]. Our study showed that STG vaccinated rats significantly induced CD4^+^ and CD8^+^ T cell populations compared to FKC and PBS control groups. Jawale et al [25] demonstrated that BG vaccine-induced CD4^+^ and CD8^+^ T-cells were involved in protection against *Salmonella* infections. However, recent studies have suggested that B-cell and T-cell immune responses are equally important for protective immunity against *S*. *typhimurium* [53,54].

Recently, genetically inactivated BG vaccines decreased bacterial load in tissue homogenates and provided protection against *Salmonella* infections [25,46]. Similarly, immunization with the chemically-induced SEG vaccine reduced bacterial clearance in liver, lung, spleen, and kidney and protected rats from *S*. *enteritidis* challenge [17]. In a previous study with *Salmonella* live vaccines, aromatic amino acid-dependent, streptomycin dependent and *galE* mutants [55–58] effectively induced protective immunity against a homologous challenge. However, these vaccines have limited potential to induce protective immunity against heterologous challenge. Effective *Salmonella* vaccination in livestock needs the induction of homologous and heterologous protective immunity. Based on these results, vaccination with the STG vaccine strongly induced protective immune responses and protected experimental animals from *S*. *typhimurium* and *S*. *enteritidis* infections.

## Conclusion

In conclusion, vaccination with the STG vaccine induced higher levels of humoral and cell-mediated immunity than the FKC vaccine and exhibited a significant reduction in bacterial colonization against *S*. *typhimurium* and *S*. *enteritidis* strains. The potential of chemically-induced BG to heterologous protection is now being evaluated. Moreover, the results obtained from this study suggest that the envelope antigen expression could be an important component of inducing cross-protective immune responses to the *S*. *enteritidis* strain. Therefore, the STG vaccine could be a promising candidate for the development of inactivated vaccines against *Salmonella* infections.

## Supporting information

S1 Table Primers sequences of the targeted genes in real-time PCR. (DOCX)

## References

[1] Hohmann EL. Nontyphoidal salmonellosis. Clin Infect Dis 2001;32:263–9. doi: 10.1086/318457 11170916 10.1086/318457

[2] Rabsch W, Tschäpe H, Bäumler AJ. Non-typhoidal salmonellosis: emerging problems. Microbes Infect. 2001;3: 237–247. 11358718 10.1016/s1286-4579(01)01375-2

[3] Majowicz SE, Musto J, Scallan E, Angulo FJ, Kirk M, O'Brien SJ, et al The global burden of nontyphoidal *Salmonella* gastroenteritis. Clin Infect Dis. 2010;50: 882–9. doi: 10.1086/650733 20158401 10.1086/650733

[4] Breuil J, Berger N, Dublanchet A, the College BVH. Sensibilite´ aux antibiotiques de 2800 souches de Salmonelles et Shigelles isole´es en France en 1994. Med Mal Infect. 1996;26: 420–5. 17292313 10.1016/s0399-077x(96)80186-7

[5] Casin I, Brisabois A, Berger N, Breuil J, Collatz E. Resistance phenotypes and genotypes of 182 ampicillin-resistant *Salmonella typhymurium* strains of human and animal origin. Med Mal Infect. 1996;26: 426–30. 17292314 10.1016/s0399-077x(96)80187-9

[6] Martel JL, Chaslus-Dancla E, Coudert M, Lafont JP. Evolution of antimicrobial susceptibility in salmonellas from bovine origin in France. Med Mal Infect. 1996;26: 415–9. 17292312 10.1016/s0399-077x(96)80185-5

[7] Stubbs AD, Hickman-Brenner FW, Cameron DN, Farmer III JJ. Differentiation of *Salmonella enteritidis* phage type 8 strains: evaluation of three additional phage typing systems, plasmid profiles, antibiotic susceptibility patterns, and biotyping. J Clin Microbiol. 1994;32: 199–201. 8126179 10.1128/jcm.32.1.199-201.1994 PMC262995

[8] Harrison JA, Villarreal-Ramos B, Mastroeni P, Demarco de Hormaeche R, Hormaeche CE. Correlates of protection induced by live *Aro*-*Salmonella typhimurium* vaccines in the murine typhoid model. Immunology. 1997;90: 618–25. 9176117 10.1046/j.1365-2567.1997.00158.x PMC1456680

[9] Hormaeche CE, Joysey HS, Desilva L, Izhar M, Stocker BAD. Immunity conferred by *AroA*- *Salmonella* live vaccines. Microb Pathog. 1991;10: 149–58. 1890952 10.1016/0882-4010(91)90075-l

[10] Hormaeche CE, Mastroeni P, Harrison JA, Demarco de Hormaeche R, Svenson S, Stocker BAD. Protection against oral challenge three months after i.v. immunization of BALB/c mice with live *Aro Salmonella typhimurium* and *Salmonella enteritidis* vaccines is serotype (species)- dependent and only partially determined by the main LPS O antigen. Vaccine. 1996;14: 251–9. 8744548 10.1016/0264-410x(95)00249-z

[11] Cooper GL, Venables LM, Nicholas RA, Cullen GA, Hormaeche CE. Vaccination of chickens with chicken-derived *Salmonella enteritidis* phage type 4 *aroA* live oral *Salmonella* vaccines. Vaccine. 1992;10: 247–54. 1561832 10.1016/0264-410x(92)90160-l

[12] Barrow PA, Hassan JO, Berchieri A Jr. Reduction in faecal excretion of *Salmonella typhimurium* strain F98 in chickens vaccinated with live and killed *S*. *typhimurium* organisms. Epidemiol Infect. 1990;104: 413–26. 2189743 10.1017/s0950268800047439 PMC2271771

[13] Hassan JO, Curtiss R III. Development and evaluation of an experimental vaccination program using a live avirulent *Salmonella typhimurium* strain to protect immunized chickens against challenge with homologous and heterologous *Salmonella* serotypes. Infect Immun. 1994;62: 5519–27. 7960134 10.1128/iai.62.12.5519-5527.1994 PMC303297

[14] Langemann T, Koller VJ, Muhammad A, Kudela P, Mayr UB, Lubitz W. The Bacterial Ghost platform system: production and applications. Bioeng Bugs. 2010;1: 326–36. doi: 10.4161/bbug.1.5.12540 21326832 10.4161/bbug.1.5.12540 PMC3037582

[15] Kwon SR, Nam YK, Kim SK, Kim DS, Kim KH. Generation of *Edwardsiella tarda* ghosts by bacteriophage PhiX174 lysis gene *E*. Aquaculture. 2005;250: 16–21.

[16] Zhu W, Yang G, Zhang Y, Yuan J, An L. Generation of biotechnology-derived *Flavobacterium columnare* ghosts by PhiX174 gene *E*-mediated inactivation and the potential as vaccine candidates against infection in grass carp. J Biomed Biotechnol. 2012;2012: 1–8. doi: 10.1155/2012/728342 22719209 10.1155/2012/760730 PMC3376489

[17] Vinod N, Oh S, Kim S, Choi CW, Kim SC, Jung CH. Chemically induced *Salmonella enteritidis* ghosts as a novel vaccine candidate against virulent challenge in a rat model. Vaccine. 2014;32: 3249–55. doi: 10.1016/j.vaccine.2014.03.090 24721534 10.1016/j.vaccine.2014.03.090

[18] Vinod N, Oh S, Park HJ, Koo JM, Choi CW, Kim SC. Generation of a novel *Staphylococcus aureus* ghost vaccine and examination of its immunogenicity against virulent challenge in rats. Infect Immun. 2015;83: 2957–65. doi: 10.1128/IAI.00009-15 25964469 10.1128/IAI.00009-15 PMC4468543

[19] Park HJ, Oh S, Vinod N, Ji S, Noh HB, Koo JM, et al Characterization of chemically-induced bacterial ghosts (NGs) using sodium hydroxide-induced *Vibrio parahaemolyticus* ghosts (VPGs). Int J Mol Sci. 2016;17: 1904. 10.3390/ijms17111904 PMC5133902 27854308

[20] Amara AA, Salem-Bekhit MM, Alanazi FK. Sponge-like: a new protocol for preparing bacterial ghosts. Sci World J. 2013;2013: 1–7. 10.1155/2013/545741 PMC3614036 23576904

[21] Amara AA, Salem-Bekhit MM, Alanazi FK. Plackett–Burman randomization method for Bacterial Ghosts preparation form *E*. *coli* JM109. Saudi Pharm J. 2013;22: 273–9. doi: 10.1016/j.jsps.2013.06.002 25061413 10.1016/j.jsps.2013.06.002 PMC4099561

[22] Eko FO, Mayr UB, Attridge SR, Lubitz W. Characterization and immunogenicity of *Vibrio cholera* ghosts expressing toxin-coregulated pili. J Biotechnol. 2000;83: 115–23. 11000467 10.1016/s0168-1656(00)00315-1

[23] Lubitz P, Mayr UB, Lubitz W. Applications of bacterial ghosts in biomedicine. Adv Exp Med Biol. 2009;655: 159–70. doi: 10.1007/978-1-4419-1132-2_12 20047041 10.1007/978-1-4419-1132-2_12

[24] Mayr UB, Kudela P, Atrasheuskaya A, Bukin E, Ignatyev G, Lubitz W. Rectal single dose immunization of mice with *Escherichia coli* O157:H7 bacterial ghosts induces efficient humoral and cellular immune responses and protects against the lethal heterologous challenge. Microb Biotechnol. 2012;5: 283–94. doi: 10.1111/j.1751-7915.2011.00316.x 22103353 10.1111/j.1751-7915.2011.00316.x PMC3815788

[25] Jawale CV, Chaudhari AA, Jeon BW, Nandre RM, Lee JH. Characterization of a novel inactivated *Salmonella enterica* Serovar Enteritidis vaccine candidate generated using a modified cI857/λ PR/gene *E* expression system. Infect Immun. 2012;80: 1502–09. doi: 10.1128/IAI.06264-11 22290147 10.1128/IAI.06264-11 PMC3318409

[26] Chaudhari AA, Jawale CV, Kim SW, Lee JH. Construction of a *Salmonella gallinarum* ghost as a novel inactivated vaccine candidate and its protective efficacy against fowl typhoid in chickens. Vet Res. 2012;43: 1–11. doi: 10.1186/1297-9716-43-1 22620989 10.1186/1297-9716-43-44 PMC3413521

[27] Hougen HP, Jensen ET, Klausen B. Experimental *Salmonella typhimurium* infections in rats. I: Influence of thymus on the course of infection. APMIS. 1989;97: 825–32. 2675938

[28] Havelaar AH, Garssen J, Takumi K, Koedam MA, Dufrenne JB, van Leusden FM, de La Fonteyne L, Bousema JT, Vos JG. A rat model for dose-response relationships of *Salmonella Enteritidis* infection. J Appl Microbiol. 2001;91: 442–52. 11556909 10.1046/j.1365-2672.2001.01399.x

[29] Redman TK, Harmon CC, Lallone RL, Michalek SM. Oral immunization with recombinant *Salmonella typhimurium* expressing surface protein antigen A of *Streptococcus sobrinus*: dose response and induction of protective humoral responses in rats. Infect Immun. 1995;63: 2004–11. 7729915 10.1128/iai.63.5.2004-2011.1995 PMC173257

[30] Hossain MMM, Ehsan A, Rahman MA, Chowdhury MBR, Haq M. Responses of monosex nile tilapia (*Oreochromis niloticus*) to intraperitoneal challenge by *Streptococcus iniae* after vaccination with ghosts of the bacterium. Bangl Vet. 2012;29: 31–7.

[31] Palva ET, Mäkelä PH. Lipopolysaccharide heterogeneity in *Salmonella typhimurium* analyzed by sodium dodecyl sulfate polyacrylamide gel electrophoresis. Eur J Biochem. 1980;107: 137–43. 6995111 10.1111/j.1432-1033.1980.tb04634.x

[32] Palva ET. Major outer membrane protein in *Salmonella typhimurium* induced by maltose. J Bacteriol. 1978;136: 286–94. 361696 10.1128/jb.136.1.286-294.1978 PMC218659

[33] Richter-Dahlfors A, Buchan AM, Finlay BB. Murine salmonellosis studied by confocal microscopy: *Salmonella typhimurium* resides intracellularly inside macrophages and exerts a cytotoxic effect on phagocytes *in vivo*. J Exp Med. 1997;186: 569–80. 9254655 10.1084/jem.186.4.569 PMC2199036

[34] Rescigno M, Urbano M, Valzasina B, Francolini M, Rotta G, Bonasio R, et al, Dendritic cells express tight junction proteins and penetrate gut epithelial monolayers to sample bacteria. Nat Immunol. 2001;2: 361–7. doi: 10.1038/86373 11276208 10.1038/86373

[35] Yrlid U, Svensson M, Kirby A, Wick MJ. Antigen-presenting cells and anti-Salmonella immunity. Microbes Infect. 2001;3: 1239–48. 11755412 10.1016/s1286-4579(01)01484-8

[36] Mastroeni P. Immunity to systemic Salmonella infections. Curr Mol Med. 2002;2: 393–406. 12108950 10.2174/1566524023362492

[37] Zhao C, Wood MW, Galyov EE, Höpken UE, Lipp M, Bodmer HC, Tough DF, Carter RW. *Salmonella typhimurium* infection triggers dendritic cells and macrophages to adopt disyinct migration patterns *in vivo*. Eur J Immunol. 2006;36: 2939–50. doi: 10.1002/eji.200636179 17048271 10.1002/eji.200636179

[38] Seid RC, Sadoff JC. Preparation and characterization of detoxified lipopolysaccharide-protein conjugates. J Biol Chem. 1981;256: 7305–10. 6166612

[39] Von Eschen KB, Rudbach JA. Antibody responses of mice to alkaline detoxified lipopolysaccharide. J Immunol. 1976;116: 8–11. 1107425

[40] ČiŽnár I, Shands JW. Effect of alkali on the immunological reactivity of lipopolysaccharide from *Salmonella typhimurium*. Infect Immun. 1970;2: 549–55. 16557876 10.1128/iai.2.5.549-555.1970 PMC416049

[41] Svensson M, Johansson C, Wick MJ. *Salmonella typhimurium*-induced cytokine production and surface molecule expression by murine macrophages. Microb Pathog. 2001;31: 91–102. doi: 10.1006/mpat.2001.0448 11453704 10.1006/mpat.2001.0448

[42] Rosenberger CM, Scott MG, Gold MR, Hancock REW, Finlay BB. *Salmonella typhimurium* infection and lipopolysaccharide stimulation induce similar changes in macrophage gene expression. J Immunol. 2000;164: 5894–904. 10820271 10.4049/jimmunol.164.11.5894

[43] Mastroeni P, Vazquez-Torres A, Fang FC, Xu Y, Khan S, Hormaeche CE, et al Antimicrobial actions of the NADPH phagocyte oxidase and inducible nitric oxide synthase in experimental salmonellosis. II. Effects on microbial proliferation and host survival in vivo. J Exp Med. 2000;192: 237–48. 10899910 10.1084/jem.192.2.237 PMC2193252

[44] Vazquez-Torres A, Fang FC. Oxygen-dependent anti-*Salmonella* activity of macrophages. Trends Microbiol. 2001;9: 29–33. 11166240 10.1016/s0966-842x(00)01897-7

[45] Vazquez-Torres A, Fantuzzi G, Edwards CK III, Dinarello CA, Fang FC. Defective localization of the NADPH phagocyte oxidase to *Salmonella*-containing phagosomes in tumor necrosis factor p55 receptor-deficient macrophages. Proc Natl Acad Sci USA. 2001;98: 2561–5. doi: 10.1073/pnas.041618998 11226278 10.1073/pnas.041618998 PMC30177

[46] Peng W, Si W, Yin L, Liu H, Yu S, Liu S, et al *Salmonella enteritidis* ghost vaccine induces effective protection against lethal challenge in specific-pathogen-free chicks. Immunobiology. 2011;216: 558–65. doi: 10.1016/j.imbio.2010.10.001 21247655 10.1016/j.imbio.2010.10.001

[47] Hofstra H, Danbert J. Major outer membrane proteins: common antigens in Enterobacteriaceae species. J Gen Microbiol. 1980;119: 123–31. doi: 10.1099/00221287-119-1-123 6157776 10.1099/00221287-119-1-123

[48] Hu M, Zhang Y, Xie F, Li G, Li J, Si W, et al Protection of piglets by a *Haemophilus parasuis* ghost vaccine against homologous challenge. Clin Vaccine Immunol. 2013;20: 795–802. doi: 10.1128/CVI.00676-12 23536691 10.1128/CVI.00676-12 PMC3675978

[49] Wang X, Lu C. Mice orally vaccinated with *Edwardsiella tarda* ghosts are significantly protected against infection. Vaccine. 2009;27: 1571–8. doi: 10.1016/j.vaccine.2009.01.002 19166892 10.1016/j.vaccine.2009.01.002

[50] Schat K. Cell-mediated immune effector function in chickens. Poult Sci. 1994;73: 1077–81. 7937468 10.3382/ps.0731077

[51] Salerno-Goncalves R, Pasetti MF, Sztein MB. Characterization of CD8(+) effector T cell responses in volunteers immunized with *Salmonella enterica serovar Typhi* strain Ty21a *typhoid vaccine*. J Immunol. 2002;169: 2196–203. 12165550 10.4049/jimmunol.169.4.2196

[52] Lillehoj H, Okamura M. Host immunity and vaccine development to *coccidian* and *salmonella* infections in chickens. J Poult Sci. 2003;40: 151–93.

[53] Miller SI, Kukral AM, Mekalanos JJ. A two-component system (phoP-phoQ) controls *Salmonella typhimurium* virulence. Proc Natl Acad Sci USA. 1989;86: 5054–8. 2544889 10.1073/pnas.86.13.5054 PMC297555

[54] Mittrucker HW, Kaufmann SHF. Immune response to infection with *Salmonella typhimurium* in mice. J Leukoc Biol. 2000;67: 457–63. 10770276 10.1002/jlb.67.4.457

[55] Da Roden L, Smith BP, Spier SJ, Dilling GW. Effect of calf age and *Salmonella* bacterin type on ability to produce immunoglobulins directed against *Salmonella* whole cells or lipopolysaccharide. Am J Vet Res. 1992;53: 1895–9. 1456538

[56] Habasha FG, Smith BP, Schwartz L, Ardans A, Reina-Guerra M. Correlation of macrophage migration-inhibition factor and protection from challenge exposure in calves vaccinated with *Salmonella typhimurium*. Am J Vet Res. 1985;46: 1415–21. 3896069

[57] Smith BP, Dilling GW, Da Roden L, Stocker BA. Vaccination of calves with orally administered aromatic-dependent *Salmonella dublin*. Am J Vet Res. 1993;54: 1249–55. 8214891

[58] Villarreal-Ramos B, Manser J, Collins RA, Dougan G, Chatfield SN, Howard CJ. Immune responses in calves immunised orally or subcutaneously with a live *Salmonella typhimurium* aro vaccine. Vaccine. 1998;16: 45–54. 9607008 10.1016/s0264-410x(97)00156-4

