# Supplementary material for: A Salmonella typhimurium ghost vaccine induces cytokine expression in vitro and immune responses in vivo and protects rats against homologous and heterologous challenges
Source: PLoS One. 2017 Sep 29;12(9):e0185488. doi: 10.1371/journal.pone.0185488 (PMC5621678; doi:10.1371/journal.pone.0185488)
Supplement: S1 Table — (DOCX) [file pone.0185488.s001.docx]

**S1 Table:** Primer sequences of the targeted genes in real-time RT-qPCR.

| **Gene** | **Orientation** | **Primer Sequences (5′–3′)** |
| --- | --- | --- |
| TNF-α | forward | ATGAGCACAGAA AGCATGATCCG |
|  | reverse | GCTGAGACATAGGCACCGC |
| Il-1β | forward | ATGGCAACTGTTCCTGAACTCAACT |
|  | reverse | AGTAGCCCTTCATCTTTTGGGG |
| IL-6 | forward | ATGAAGTTCCTCTCTGCAAGAGACT |
|  | reverse | GTCTCCTCTCCGGACTTGTGA |
| IL-10 | forward | ATGCCTGGCTCAGCACTGCTA |
|  | reverse | CTGGGAAGTGGGTGCAGTTATTG |
| iNOS | forward | ATGAACCCCAAGAGTTTGACCAGA |
|  | reverse | GGAGCCATAATACTGGTTGATGAAC |
| GAPDH | forward | ATGGTGAAGGTCGGTGTGAACG |
|  | reverse | CAATGAAGGGGTCGTTGATGGC |
